# 3,15-Dimeth­oxy-10-methyl­tricyclo­[9.4.0.0^2,7^]penta­deca-1(11),2(7),3,5,9,12,14-heptaen-8-one

**DOI:** 10.1107/S160053681103100X

**Published:** 2011-08-11

**Authors:** Yaomin Zhu, Jianfei Yang, Xianfei Li, Le Zhou

**Affiliations:** a School of Materials Science and Engineering, Henan University of Science & Technology 471022, People’s Republic of China; b College of Science, Northwest A&F University, Yangling 712100, People’s Republic of China

## Abstract

The title mol­ecule, C_18_H_16_O_3_, contains three fused rings, of which the seven-membered cyclo­hept-2-enone ring has a screw-boat conformation. The two meth­oxy­phenyl rings make a dihedral angle of 50.4 (2)°. In the crystal, mol­ecules are linked by inter­molecular C—H⋯O hydrogen bonds, leading to a three-dimensional supra­molecular architecture.

## Related literature

The title compound was obtained through an aldol condensation reaction. For general background to aldol reactions, see: Machajewski & Wong (2000 ▶); Nelson (1998 ▶). For structures with C—H⋯O hydrogen bonds, see: Broder *et al.* (2002 ▶); Senthil Kumar *et al.* (2006 ▶).
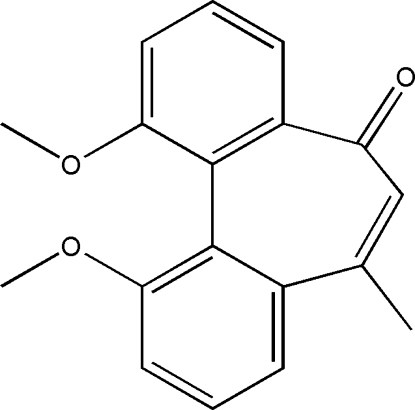


## Experimental

### 

#### Crystal data

C_18_H_16_O_3_
*M* *_r_* = 280.31
Orthorhombic, 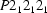

*a* = 7.6615 (10) Å
*b* = 12.2005 (16) Å
*c* = 15.545 (2) Å
*V* = 1453.1 (3) Å^3^
*Z* = 4
Mo *K*α radiation
μ = 0.09 mm^−1^
*T* = 295 K
0.43 × 0.31 × 0.17 mm

#### Data collection

Bruker SMART CCD area detector diffractometer
Absorption correction: multi-scan (*SADABS*; Sheldrick, 1996 ▶) *T*_min_ = 0.964, *T*_max_ = 0.986
11119 measured reflections
2708 independent reflections
2083 reflections with *I* > 2σ(*I*)
*R*_int_ = 0.034

#### Refinement

*R*[*F* ^2^ > 2σ(*F* ^2^)] = 0.037
*wR*(*F* ^2^) = 0.090
*S* = 1.09
2708 reflections
193 parameters
H-atom parameters constrained
Δρ_max_ = 0.10 e Å^−3^
Δρ_min_ = −0.13 e Å^−3^

### 

Data collection: *SMART* (Bruker, 2004 ▶); cell refinement: *SAINT* (Bruker, 2004 ▶); data reduction: *SAINT*; program(s) used to solve structure: *SHELXS97* (Sheldrick, 2008 ▶); program(s) used to refine structure: *SHELXL97* (Sheldrick, 2008 ▶); molecular graphics: *SHELXTL* (Sheldrick, 2008 ▶); software used to prepare material for publication: *SHELXTL*.

## Supplementary Material

Crystal structure: contains datablock(s) global, I. DOI: 10.1107/S160053681103100X/zj2016sup1.cif

Structure factors: contains datablock(s) I. DOI: 10.1107/S160053681103100X/zj2016Isup2.hkl

Supplementary material file. DOI: 10.1107/S160053681103100X/zj2016Isup3.cml

Additional supplementary materials:  crystallographic information; 3D view; checkCIF report

## Figures and Tables

**Table 1:** Hydrogen-bond geometry (Å, °)

*D*—H⋯*A*	*D*—H	H⋯*A*	*D*⋯*A*	*D*—H⋯*A*
C13—H13*B*⋯O3^i^	0.96	2.40	3.349 (3)	171
C10—H10⋯O1^ii^	0.93	2.58	3.283 (2)	133

Symmetry codes: (i) 
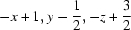
; (ii) 
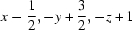
.

**Fig. 1.**
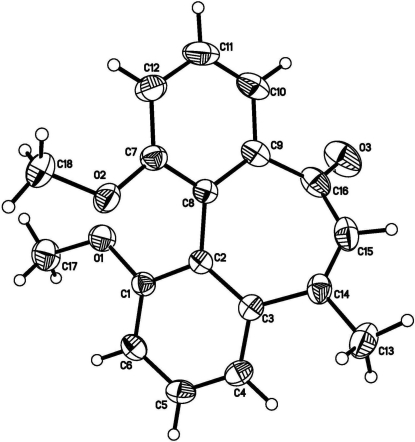
View of the title molecular structure with atom numbering scheme and 50% probability displacement ellipsoids for non-hydrogen atoms.

**Fig. 2.**
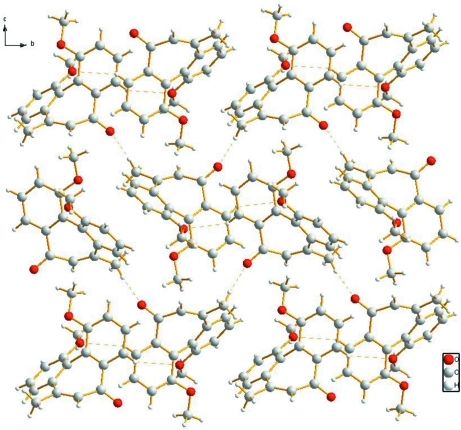
View of three-dimensional structure (C—H···O hydrogen bonds are represented as dashed lines).

## supplementary crystallographic information

### Comment

Direct aldol reactions provide an atom-economical approach to create the β-hydroxy carbonyl structural unit found in many natural products and drugs (Machajewski *et al.* 2000; Nelson, 1998.). In our study, we were interested to the intramolecular aldol condensation reaction. To our surprise, the resulting aldol adducts are further dehydrated to afford an enone compound. The title molecule is built up from three fused rings including two phenyl rings and one seven-membered ring (Fig. 1). The non aromatic seven-membered ring has a screw boat conformation. The two methoxyphenyl rings make dihedral angles of 50.4 (2) Å. In the crystal structure, the weak intermolecular C—H···O hydrogen bonds are observed. Thus, molecules are linked to each other by intermolecular C13—H13B···O3 hydrogen bonds (C13···O3 = 3.349 (3) Å), resulting in a one-dimensional chain. The chains are further connected through the formation of intermolecular C10—H10···O1 hydrogen bonds (C10···O1 = 3.283 (2) Å), leading to a three-dimensional supmolecular architecture, as shown in Fig. 2.

### Experimental

2,2-dimethoxy-6,6-diacetyl-1,1-biphenyl (298 mm g, 1 mmol) was added to a solution of CH_3_CH_2_ONa (6.8 mg, 0.1 mmol) and enthanol (5 ml) at room temperature. The mixture was stirred, monitored by TLC. After 8 h, the mixture was extracted by ethyl acetate (3× 15 ml). The resulting solvent was removed *in vacuo* to yield the crude product. Purification by silica gel chromatography using 100 ~200 mesh ZCX II eluted by hexane-ethyl acetate (3:1, *v*/*v*) gave the yellow solid (196 mg, yield 70%). The crystalline compound was obtained through the slow volatilization of ethyl acetate containing the title compound.

### Refinement

All H atoms were positioned geometrically and treated as riding, with C—H bond lengths constrained to 0.93 Å (aromatic CH), 0.93 Å (methylene CH_2_), or 0.96 Å (methyl CH_3_), and with *U*ĩso~(H) = 1.2Ueq(C) or 1.5Ueq(methyl and methylene C).

### Crystal data

**Table:**

C_18_H_16_O_3_	*F*(000) = 592
*M**_r_* = 280.31	*D*_x_ = 1.281 Mg m^−^^3^
Orthorhombic, *P*2_1_2_1_2_1_	Mo *K*α radiation, λ = 0.71073 Å
Hall symbol: P 2ac 2ab	Cell parameters from 2351 reflections
*a* = 7.6615 (10) Å	θ = 2.6–22.6°
*b* = 12.2005 (16) Å	µ = 0.09 mm^−^^1^
*c* = 15.545 (2) Å	*T* = 295 K
*V* = 1453.1 (3) Å^3^	Block, yellow
*Z* = 4	0.43 × 0.31 × 0.17 mm

### Data collection

**Table:**

Bruker SMART CCD area detector diffractometer	2708 independent reflections
Radiation source: fine-focus sealed tube	2083 reflections with *I* > 2σ(*I*)
graphite	*R*_int_ = 0.034
φ and ω scans	θ_max_ = 25.5°, θ_min_ = 2.6°
Absorption correction: multi-scan (*SADABS*; Sheldrick, 1996)	*h* = −9→9
*T*_min_ = 0.964, *T*_max_ = 0.986	*k* = −14→14
11119 measured reflections	*l* = −18→18

### Refinement

**Table:**

Refinement on *F*^2^	Primary atom site location: structure-invariant direct methods
Least-squares matrix: full	Secondary atom site location: difference Fourier map
*R*[*F*^2^ > 2σ(*F*^2^)] = 0.037	Hydrogen site location: inferred from neighbouring sites
*wR*(*F*^2^) = 0.090	H-atom parameters constrained
*S* = 1.09	*w* = 1/[σ^2^(*F*_o_^2^) + (0.0385*P*)^2^ + 0.0759*P*] where *P* = (*F*_o_^2^ + 2*F*_c_^2^)/3
2708 reflections	(Δ/σ)_max_ < 0.001
193 parameters	Δρ_max_ = 0.10 e Å^−^^3^
0 restraints	Δρ_min_ = −0.13 e Å^−^^3^

### Special details

**Table:**

Geometry. All e.s.d.'s (except the e.s.d. in the dihedral angle between two l.s. planes)are estimated using the full covariance matrix. The cell e.s.d.'s are takeninto account individually in the estimation of e.s.d.'s in distances, anglesand torsion angles; correlations between e.s.d.'s in cell parameters are onlyused when they are defined by crystal symmetry. An approximate (isotropic)treatment of cell e.s.d.'s is used for estimating e.s.d.'s involving l.s. planes.
Refinement. Refinement of *F*^2^ against ALL reflections. The weighted *R*-factor *wR* and goodness of fit *S* are based on *F*^2^, conventional *R*-factors *R* are based on *F*, with *F* set to zero for negative *F*^2^. The threshold expression of *F*^2^ > σ(*F*^2^) is used only for calculating *R*-factors(gt) *etc*. and is not relevant to the choice of reflections for refinement. *R*-factors based on *F*^2^ are statistically about twice as large as those based on *F*, and *R*- factors based on ALL data will be even larger.

### Fractional atomic coordinates and isotropic or equivalent isotropic displacement parameters (Å^2^)

**Table:**

	*x*	*y*	*z*	*U*_iso_*/*U*_eq_	
O1	0.90746 (18)	0.51793 (11)	0.45436 (10)	0.0617 (4)	
O2	0.6310 (2)	0.46929 (11)	0.35471 (8)	0.0590 (4)	
O3	0.4247 (3)	0.68747 (15)	0.67899 (12)	0.0975 (6)	
C1	0.8412 (3)	0.43948 (14)	0.50732 (12)	0.0435 (5)	
C2	0.6641 (2)	0.45458 (13)	0.53147 (11)	0.0391 (4)	
C3	0.5949 (3)	0.38457 (15)	0.59460 (11)	0.0423 (5)	
C4	0.6961 (3)	0.29640 (16)	0.62432 (12)	0.0506 (5)	
H4	0.6490	0.2483	0.6645	0.061*	
C5	0.8624 (3)	0.27996 (16)	0.59543 (14)	0.0544 (5)	
H5	0.9256	0.2196	0.6147	0.065*	
C6	0.9376 (3)	0.35217 (15)	0.53789 (14)	0.0516 (5)	
H6	1.0522	0.3421	0.5199	0.062*	
C7	0.5694 (3)	0.55704 (15)	0.39995 (13)	0.0478 (5)	
C8	0.5662 (2)	0.54490 (14)	0.48981 (12)	0.0415 (4)	
C9	0.4791 (3)	0.62605 (15)	0.53714 (14)	0.0502 (5)	
C10	0.4157 (3)	0.72015 (16)	0.49742 (18)	0.0658 (6)	
H10	0.3607	0.7740	0.5300	0.079*	
C11	0.4340 (3)	0.73350 (19)	0.41045 (19)	0.0756 (7)	
H11	0.3966	0.7980	0.3844	0.091*	
C12	0.5077 (3)	0.65175 (18)	0.36140 (17)	0.0657 (6)	
H12	0.5160	0.6602	0.3021	0.079*	
C13	0.3278 (3)	0.3040 (2)	0.66884 (15)	0.0734 (7)	
H13A	0.2127	0.3251	0.6867	0.110*	
H13B	0.3899	0.2736	0.7169	0.110*	
H13C	0.3195	0.2501	0.6240	0.110*	
C14	0.4239 (3)	0.40290 (18)	0.63578 (12)	0.0519 (5)	
C15	0.3608 (3)	0.5025 (2)	0.65305 (13)	0.0634 (6)	
H15	0.2579	0.5040	0.6848	0.076*	
C16	0.4316 (3)	0.6103 (2)	0.62877 (14)	0.0624 (6)	
C17	1.0812 (3)	0.5087 (2)	0.42411 (17)	0.0778 (7)	
H17A	1.0926	0.4431	0.3904	0.117*	
H17B	1.1596	0.5054	0.4722	0.117*	
H17C	1.1091	0.5712	0.3893	0.117*	
C18	0.6894 (4)	0.4865 (3)	0.26972 (15)	0.0946 (10)	
H18A	0.7702	0.5466	0.2687	0.142*	
H18B	0.5914	0.5032	0.2335	0.142*	
H18C	0.7462	0.4215	0.2491	0.142*	

### Atomic displacement parameters (Å^2^)

**Table:**

	*U*^11^	*U*^22^	*U*^33^	*U*^12^	*U*^13^	*U*^23^
O1	0.0433 (8)	0.0547 (8)	0.0872 (10)	−0.0025 (7)	0.0133 (8)	0.0130 (8)
O2	0.0767 (10)	0.0580 (8)	0.0422 (8)	0.0003 (8)	0.0094 (7)	0.0039 (7)
O3	0.1176 (15)	0.0921 (12)	0.0829 (12)	0.0259 (12)	−0.0124 (11)	−0.0455 (11)
C1	0.0437 (12)	0.0397 (9)	0.0472 (11)	−0.0009 (9)	0.0022 (9)	−0.0023 (8)
C2	0.0390 (10)	0.0380 (9)	0.0403 (10)	−0.0017 (8)	−0.0028 (8)	−0.0037 (8)
C3	0.0445 (11)	0.0457 (10)	0.0367 (9)	−0.0050 (9)	−0.0059 (9)	−0.0040 (8)
C4	0.0575 (14)	0.0501 (11)	0.0442 (12)	−0.0052 (10)	−0.0081 (10)	0.0055 (9)
C5	0.0585 (14)	0.0481 (11)	0.0566 (12)	0.0082 (10)	−0.0117 (11)	0.0036 (10)
C6	0.0429 (12)	0.0533 (11)	0.0586 (13)	0.0059 (10)	−0.0017 (11)	−0.0056 (10)
C7	0.0465 (12)	0.0436 (10)	0.0533 (12)	−0.0009 (10)	−0.0003 (10)	0.0048 (9)
C8	0.0371 (10)	0.0393 (9)	0.0481 (11)	−0.0021 (8)	−0.0021 (9)	−0.0003 (8)
C9	0.0426 (12)	0.0455 (10)	0.0626 (14)	0.0007 (9)	−0.0098 (10)	−0.0103 (10)
C10	0.0577 (14)	0.0449 (12)	0.0949 (19)	0.0099 (11)	−0.0053 (13)	−0.0067 (12)
C11	0.0739 (18)	0.0526 (13)	0.100 (2)	0.0139 (13)	−0.0096 (16)	0.0202 (14)
C12	0.0654 (15)	0.0643 (14)	0.0673 (16)	0.0037 (12)	−0.0007 (13)	0.0209 (13)
C13	0.0571 (15)	0.0989 (18)	0.0641 (15)	−0.0115 (14)	0.0047 (12)	0.0218 (14)
C14	0.0446 (12)	0.0717 (14)	0.0392 (11)	0.0011 (11)	−0.0005 (10)	−0.0001 (10)
C15	0.0490 (13)	0.0945 (18)	0.0467 (12)	0.0074 (13)	0.0045 (11)	−0.0071 (12)
C16	0.0564 (14)	0.0690 (14)	0.0616 (14)	0.0134 (12)	−0.0114 (12)	−0.0210 (12)
C17	0.0504 (14)	0.0781 (16)	0.105 (2)	−0.0057 (13)	0.0223 (14)	0.0109 (15)
C18	0.126 (3)	0.108 (2)	0.0490 (13)	0.030 (2)	0.0240 (15)	0.0192 (14)

### Geometric parameters (Å, °)

**Table:**

O1—C1	1.361 (2)	C9—C16	1.483 (3)
O1—C17	1.416 (2)	C10—C11	1.369 (3)
O2—C7	1.365 (2)	C10—H10	0.9300
O2—C18	1.411 (3)	C11—C12	1.376 (3)
O3—C16	1.225 (2)	C11—H11	0.9300
C1—C6	1.381 (3)	C12—H12	0.9300
C1—C2	1.419 (3)	C13—C14	1.504 (3)
C2—C3	1.405 (2)	C13—H13A	0.9600
C2—C8	1.482 (2)	C13—H13B	0.9600
C3—C4	1.404 (3)	C13—H13C	0.9600
C3—C14	1.475 (3)	C14—C15	1.335 (3)
C4—C5	1.366 (3)	C15—C16	1.472 (3)
C4—H4	0.9300	C15—H15	0.9300
C5—C6	1.381 (3)	C17—H17A	0.9600
C5—H5	0.9300	C17—H17B	0.9600
C6—H6	0.9300	C17—H17C	0.9600
C7—C12	1.385 (3)	C18—H18A	0.9600
C7—C8	1.405 (3)	C18—H18B	0.9600
C8—C9	1.402 (3)	C18—H18C	0.9600
C9—C10	1.391 (3)		
			
C1—O1—C17	119.71 (17)	C10—C11—H11	119.9
C7—O2—C18	118.37 (17)	C12—C11—H11	119.9
O1—C1—C6	123.45 (19)	C11—C12—C7	120.3 (2)
O1—C1—C2	115.18 (16)	C11—C12—H12	119.8
C6—C1—C2	121.35 (18)	C7—C12—H12	119.8
C3—C2—C1	117.83 (16)	C14—C13—H13A	109.5
C3—C2—C8	124.46 (17)	C14—C13—H13B	109.5
C1—C2—C8	117.70 (16)	H13A—C13—H13B	109.5
C4—C3—C2	119.13 (18)	C14—C13—H13C	109.5
C4—C3—C14	117.64 (18)	H13A—C13—H13C	109.5
C2—C3—C14	123.12 (17)	H13B—C13—H13C	109.5
C5—C4—C3	121.30 (19)	C15—C14—C3	123.2 (2)
C5—C4—H4	119.3	C15—C14—C13	118.9 (2)
C3—C4—H4	119.3	C3—C14—C13	117.48 (19)
C4—C5—C6	120.54 (19)	C14—C15—C16	128.9 (2)
C4—C5—H5	119.7	C14—C15—H15	115.6
C6—C5—H5	119.7	C16—C15—H15	115.6
C1—C6—C5	119.47 (19)	O3—C16—C15	120.5 (2)
C1—C6—H6	120.3	O3—C16—C9	121.5 (2)
C5—C6—H6	120.3	C15—C16—C9	116.94 (18)
O2—C7—C12	123.33 (19)	O1—C17—H17A	109.5
O2—C7—C8	115.82 (16)	O1—C17—H17B	109.5
C12—C7—C8	120.8 (2)	H17A—C17—H17B	109.5
C9—C8—C7	117.10 (17)	O1—C17—H17C	109.5
C9—C8—C2	122.44 (17)	H17A—C17—H17C	109.5
C7—C8—C2	120.26 (17)	H17B—C17—H17C	109.5
C10—C9—C8	121.1 (2)	O2—C18—H18A	109.5
C10—C9—C16	116.6 (2)	O2—C18—H18B	109.5
C8—C9—C16	121.94 (18)	H18A—C18—H18B	109.5
C11—C10—C9	120.0 (2)	O2—C18—H18C	109.5
C11—C10—H10	120.0	H18A—C18—H18C	109.5
C9—C10—H10	120.0	H18B—C18—H18C	109.5
C10—C11—C12	120.2 (2)		
			
C17—O1—C1—C6	3.6 (3)	C3—C2—C8—C7	−132.37 (19)
C17—O1—C1—C2	−177.90 (18)	C1—C2—C8—C7	49.1 (2)
O1—C1—C2—C3	−171.97 (16)	C7—C8—C9—C10	−6.8 (3)
C6—C1—C2—C3	6.6 (3)	C2—C8—C9—C10	168.16 (18)
O1—C1—C2—C8	6.6 (2)	C7—C8—C9—C16	165.73 (19)
C6—C1—C2—C8	−174.83 (16)	C2—C8—C9—C16	−19.3 (3)
C1—C2—C3—C4	−6.6 (2)	C8—C9—C10—C11	1.6 (3)
C8—C2—C3—C4	174.93 (17)	C16—C9—C10—C11	−171.3 (2)
C1—C2—C3—C14	169.41 (17)	C9—C10—C11—C12	3.1 (4)
C8—C2—C3—C14	−9.1 (3)	C10—C11—C12—C7	−2.3 (4)
C2—C3—C4—C5	2.5 (3)	O2—C7—C12—C11	174.6 (2)
C14—C3—C4—C5	−173.75 (18)	C8—C7—C12—C11	−3.2 (3)
C3—C4—C5—C6	2.1 (3)	C4—C3—C14—C15	141.1 (2)
O1—C1—C6—C5	176.20 (18)	C2—C3—C14—C15	−34.9 (3)
C2—C1—C6—C5	−2.2 (3)	C4—C3—C14—C13	−31.5 (2)
C4—C5—C6—C1	−2.2 (3)	C2—C3—C14—C13	152.51 (19)
C18—O2—C7—C12	21.8 (3)	C3—C14—C15—C16	6.9 (3)
C18—O2—C7—C8	−160.3 (2)	C13—C14—C15—C16	179.3 (2)
O2—C7—C8—C9	−170.39 (17)	C14—C15—C16—O3	−141.6 (2)
C12—C7—C8—C9	7.6 (3)	C14—C15—C16—C9	49.7 (3)
O2—C7—C8—C2	14.6 (3)	C10—C9—C16—O3	−37.9 (3)
C12—C7—C8—C2	−167.46 (18)	C8—C9—C16—O3	149.3 (2)
C3—C2—C8—C9	52.8 (3)	C10—C9—C16—C15	130.6 (2)
C1—C2—C8—C9	−125.65 (19)	C8—C9—C16—C15	−42.2 (3)

### Hydrogen-bond geometry (Å, °)

**Table:**

*D*—H···*A*	*D*—H	H···*A*	*D*···*A*	*D*—H···*A*
C13—H13B···O3^i^	0.96	2.40	3.349 (3)	171.
C10—H10···O1^ii^	0.93	2.58	3.283 (2)	133.

Symmetry codes: (i) −*x*+1, *y*−1/2, −*z*+3/2; (ii) *x*−1/2, −*y*+3/2, −*z*+1.
